# Preliminary study of 3 T-MRI native T1-mapping radiomics in differential diagnosis of non-calcified solid pulmonary nodules/masses

**DOI:** 10.1186/s12935-021-02195-1

**Published:** 2021-10-18

**Authors:** Qinqin Yan, Yinqiao Yi, Jie Shen, Fei Shan, Zhiyong Zhang, Guang Yang, Yuxin Shi

**Affiliations:** 1 Department of Radiology, Shanghai Public Health Clinical Center, Fudan University, Shanghai, 201508 China; 2 Shanghai Key Laboratory of Magnetic Resonance, East China Normal University, Shanghai, 200062 China; 3 Department of Radiology, Zhongshan Hospital, Fudan University, Shanghai, 200032 China

**Keywords:** Native T1-mapping, Radiomics feature, Pulmonary nodules, Differential diagnosis

## Abstract

**Background:**

Cumulative CT radiation damage was positively correlated with increased tumor risks. Although it has recently been known that non-radiation MRI is alternative for pulmonary imaging. There is little known about the value of MRI T1-mapping in the diagnosis of pulmonary nodules. This article aimed to investigate the value of native T1-mapping-based radiomics features in differential diagnosis of pulmonary lesions.

**Methods:**

73 patients underwent 3 T-MRI examination in this prospective study. The 99 pulmonary lesions on native T1-mapping images were segmented twice by one radiologist at indicated time points utilizing the in-house semi-automated software, followed by extraction of radiomics features. The inter-class correlation coefficient (ICC) was used for analyzing intra-observer’s agreement. Dimensionality reduction and feature selection were performed via univariate analysis, and least absolute shrinkage and selection operator (LASSO) analysis. Then, the binary logical regression (LR), support vector machine (SVM) and decision tree classifiers with the input of optimal features were selected for differentiating malignant from benign lesions. The receiver operative characteristics (ROC) curve, area under the curve (AUC), sensitivity, specificity and accuracy were calculated. Z-test was used to compare differences among AUCs.

**Results:**

107 features were obtained, of them, 19.5% (n = 21) had relatively good reliability (ICC ≥ 0.6). The remained 5 features (3 GLCM, 1 GLSZM and 1 shape features) by dimensionality reduction were useful. The AUC of LR was 0.82(95%CI: 0.67–0.98), with sensitivity, specificity and accuracy of 70%, 85% and 80%. The AUC of SVM was 0.82(95%CI: 0.67–0.98), with sensitivity, specificity and accuracy of 70, 85 and 80%. The AUC of decision tree was 0.69(95%CI: 0.49–0.87), with sensitivity, specificity and accuracy of 50, 85 and 73.3%.

**Conclusions:**

The LR and SVM models using native T1-mapping-based radiomics features can differentiate pulmonary malignant from benign lesions, especially for uncertain nodules requiring long-term follow-ups.

## Introduction

Lung cancer is the leading cause of cancer death in men aged ≥ 40 years and women aged ≥ 60 years, causing far more deaths than breast cancer, prostate cancer, etc. Although tobacco control and improved treatment methods have reduced the mortality rate of lung cancer, it is estimated that 69,410 men (about 22%) and 62,470 women (about 22%) in the United States would still die of lung cancer in 2021, ranking the first among cancer deaths, according to the latest report [1].

Approximately 3.6–24.2% of screening LDCT scans were classified as indeterminate or positive [2], while the majority (84–95%) of the positive scans were proven false-positive [3, 4]. In addition, indeterminate results require at least one follow-up examination after few months, especially for nodules with size of 4–8 mm, which need serial recurrent CT scans at interval of 3, 6 and 9 months [5]. Cumulative radiation dose from repeated CT scans should be taken in account, either physical damage or psychological stress [6, 7], in particular, an extreme threat to children, the pregnant women, and those with low immunity. Magnetic resonance imaging (MRI), safer to those special people, is an alternative tool for chest lesions, with advantages of non-radiation, multi-parameters and functional measurements. Diffusion-weighted imaging (DWI), intra-voxel incoherent motion imaging (IVIM) and dynamic contrast enhanced MRI (DCE-MRI) are useful for differential diagnosis of pulmonary nodules [8–12]. When differentiating malignancy from benign nodules, the pooled sensitivity and specificity using DWI reached 80–88 and 89–93%, respectively [13, 14]. However, those MRI scans are limited by unsatisfactory repeat-ability caused by measurement bias and frequently motion artifacts, especially for nodules less than 20 mm [15, 16].

Radiomics features, for instance, first-order features and texture features, extracted from medical imaging are able to present the inherent heterogeneity of lesions, like coarseness, which are useful in the differential diagnosis of suspicious pulmonary nodules [17–21]. The 4-radiomic features of short axis, contour, concavity, and texture had an validation test AUROC of 0.8 (accuracy = 74.3%, sensitivity = 66.7%, specificity = 75.6%) in predicting malignancy in primary nodules [22]. The accuracy using the 4 signatures, including Laws_LSL_min, Laws_SLL_energy, Laws_SSL_skewness and Laws_EEL_uniformity, in benign or malignant classification was 84%, with the sensitivity of 92.9% and the specificity of 72.7% [23]. However, the majority of researches on pulmonary nodules radiomics features are mainly rely on CT and PET/CT modalities, and there are few reports on MRI. Additionally, sequences for instance, DWI, with obvious deformation and local drift, are unfavorable for the stability and repeat-ability of radiomics features. Native T1-mapping, obtained in one breath-holding scan, can quantify the T1-value of pixels or tissue, compared to conventional T1-weight imaging, which could be a candidate for differentiating malignant from benign lesions.

Our previous trial [24] confirmed that native T1-mapping was comparable to CT for evaluating nodules morphology. Moreover, the native T1-value is potentially useful in discriminating malignancy or tuberculosis from non-tuberculosis benign lesions. However, the value of radiomics signatures based on native T1-mapping imaging in differential diagnosis of pulmonary lesions are still not well established.

In summary, we assume that radiomics features based on native T1-mapping were capable of distinguishing the malignancy from benign lesions. Thus, this prospective study intended to uncover the underlying radiomics signatures of native T1-mapping and further investigated the value of native T1-mapping in aiding to differentiate malignant from benign lesions.

## Materials and methods

### Patients

**Fig. 1:**
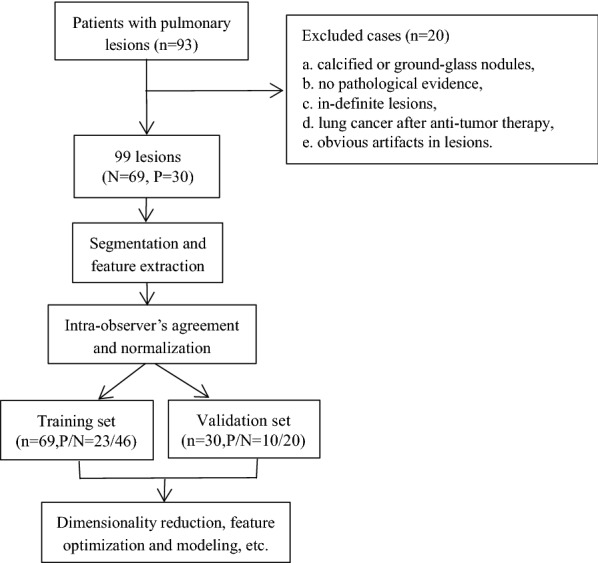
The work-flow of the patient inclusion and data analysis

93 patients with suspicious pulmonary nodules/masses underwent 3 T-MRI examinations between December 2018 and January 2020 in Shanghai Public Health Clinical Center. The exclusion criteria included: (1) calcified or ground-glass nodules, (2) no pathological evidence, (3) in-definite lesions, (4) lung cancer after anti-tumor therapy, (5) obvious artifacts in lesions because of poor breath-hold. Finally, 73 patients (54 male and 19 female, mean age, (53 ± 16) years old) with 99 non-calcified solid pulmonary nodules or masses were collected. The details of the recruited patients were listed in the Table 1 and Fig. 1. The nodules and masses consist of squamous cell carcinoma (n = 11), adenocarcinoma (n = 12), neuroendocrine carcinoma (n = 3), non-small cell lung cancer (n = 1), diffuse large B cell lymphoma (n = 1), metastasis from liver, breast and esophagus (n = 4), tuberculosis granuloma (n = 42), fungal infections (n = 10), pneumonia (n = 7), fibrosis granuloma (n = 3) and vascular granuloma (n = 5). The average size of nodules was (2.0 ± 1.1)cm (range from 0.6 to 6.8 cm). This prospective study was approved by the ethical review board of Shanghai Public Health Clinical Center (2019-S021-02).

**Table 1:** the details of the enrolled patients

	Malignant group	Benign group
Gender (F,M,n)	7, 24	12, 30
Age (mean ± SD)	62 ± 11	46 ± 18
Fever (n)	2	12
Cough (n)	12	16
Hemoptysis (n)	3	4
Chest pain (n)	7	7
Diabetes (n)	7	5
COPD (n)	0	1
Hypertension (n)	13	11
AIDS (n)	9	5
Weight loss (n)	1	2
Smoking (n)	9	3

*COPD* chronic obstructive pulmonary diseases. *AIDS* acquired immunodeficiency syndrome

^*^n means the number of individuals

### MRI scan

All patients underwent MRI examination using 3 T whole-body MR scanner (MAGNETOM Skyra, Siemens Healthcare, Erlangen, Germany) with an 18-element body wrap coil. All parameters were listed as follows: axial T1-weighted StarVIBE: TR/TE = 2.79/1.39 ms, thickness: 2 mm, field of view (FOV): 380 mm. axial T1-weight Dixon: TR/TE1/TE2 = 3.97/1.29/2.52 ms, thickness = 3 mm, FOV = 380 mm. T2-weighted fBLADE TSE: TR/TE = 1870/69 ms, thickness: 3 mm, FOV: 380 mm. T1-mapping was performed after shimming the magnetic field sequence of B1-mapping. T1-mapping: TR/TE = 5.01/2.3 ms, thickness: 4 mm, FOV: 380 mm. All patients underwent respiratory training before MRI examination.

### Nodule segmentation

**Fig. 2:**
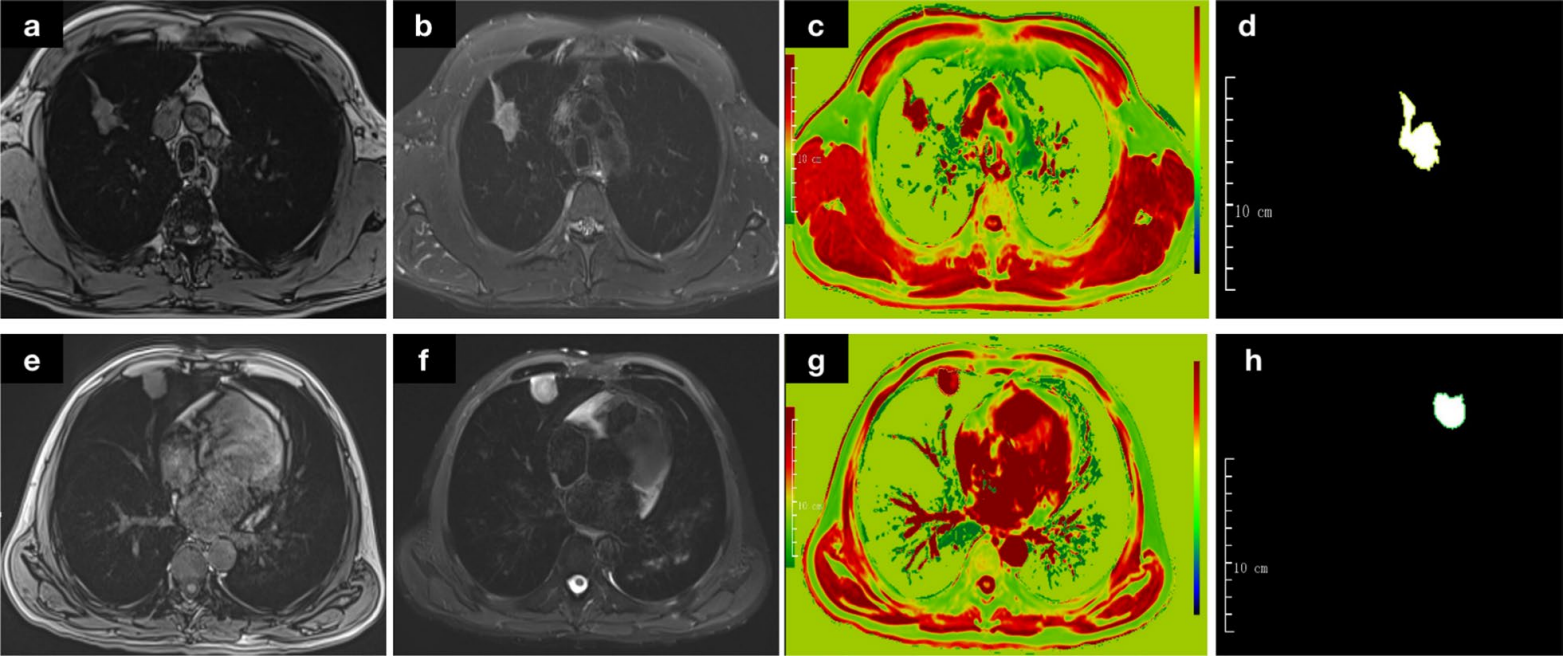
The upper row (**a**–**d**) showed an adenocarcinoma in the upper lobe of the right lung (**a** T1WI opp-phase, **b** T2WI, **c** native T1-mapping, **d** the ROI). The below row (**e**–**h**) showed a nodule with penicillium marneffei infection in the middle lobe of the right lung (**e** T1WI opp-phase, **f** T2WI, **g** native T1-mapping, **h** the ROI)

The region of interest (ROI) was segmented across all of the two-dimensional T1-mapping sections of the lesions with an in-house semi-automatic hand-annotation tool in axial view using open-source software (Multi-labe, version 1.1; Shanghai Key laboratory of Magnetic Resonance, East China Normal University, China), as shown in the Fig. 2. The radiologist was blinded to pathological diagnosis but was provided with clinical information such as age, and was also given the option to vary the window and level setting within this software to efficiently annotate the nodule. Manually and semi-automatic methods were optional for nodules annotation. The nodules or masses were annotated by the same radiologist again after 6 months for evaluating intra-observer’s agreement.

### Feature extraction and grouping

**Fig. 3:**
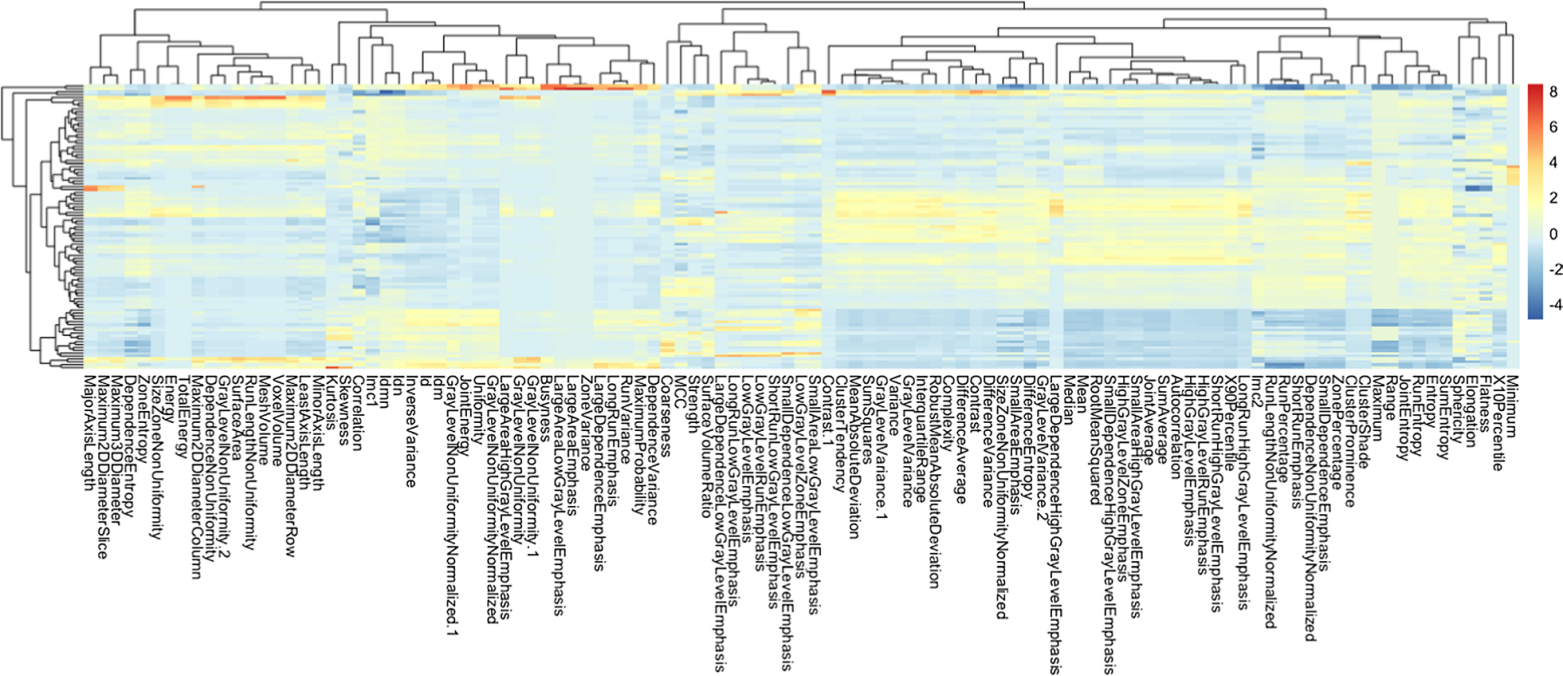
The radiomics features extracted from the native T1-mapping

A total of 107 radiomics features were extracted from the annotated mask with an open-source platform PyRadiomics (https://pyradiomics.readthedocs.io/en/latest/) [25], which enables the processing and extraction of radiomic features from medical image data and is implemented in Python. The acquired images features comprised the first-order statistics (18 features), grey-level co-occurrence matrix (GLCM, 24 features), grey-level run-length matrix (GLRLM, 16 features), grey-level size-zone matrix (GLSZM, 16 features), grey-level dependence matrix (GLDM, 14 features), neighboring gray-tone difference matrix (NGTDM, 5 features) and shape (14 features). A total of 107 feature values were normalized (mean of 0 and a standard deviation of 1), as shown in Fig. 3. This study had 99 pulmonary nodules/masses of 67 benign lesions and 32 malignancy. Among them, 69 samples (positive/negative = 23/46) were randomly selected for training set and 30 samples (positive/negative = 10/20) for the validation set based on a ratio of 7:3.

### Dimensionality reduction and radiomics feature selection

In order to avoid the over-fitting, a three-step dimensionality reduction was conducted in turns. Firstly, ICC was used for intra-observer’s agreement of radiomics features. ICC-value greater than 0.60 was considered a good agreement [26]. Secondly, t-test and wilcox-test were used for univariate analysis. P-value less than 0.05 was considered as statistical significance. Then, LASSO analysis was used for dimensionality and feature selection. tenfold cross-validation was used to reduce the over-fitting and enhance the robustness of model performance.

### Radiomics signature and models

Three classical machine methods, binary LR, SVM and decision tree were used to select optimal features and develop the model for that differential diagnosis. Multivariable binary logistic regression with backward stepwise selection was used to build a linear classifier. Then, the SVM with a Gaussian kernel was used to build a non-linear classifier. Optimizing the parameters of the SVM kernel function were realized by tenfold cross-validation, which was capable of selecting the best performing signature. The decision tree is a simple linear classifier with a sequence of questions. tenfold cross-validation and reduction of branches were applied to avoid over-fitting.

### Statistical analysis

All statistical analysis were conducted using R software (version, 4.0.3, http://r-project.org). The Shapiro–Wilk test was used to assess the normality of distributions, and the homogeneity of variance was tested using Bartlett’s test. The assessment of the optimal radiomics signature and diagnostic performance mainly relied on ROC. Accuracy, sensitivity, and specificity, were also calculated at the maximum of Youden’s index. Each radiomics signature was also assessed according to all of the metrics for the validation cohort. Z-test was used for comparisons of diagnostic performance of inter-models. P-value less than 0.5 was considered as statistical significance.

## Results

### ICCs of radiomics features

**Fig. 4:**
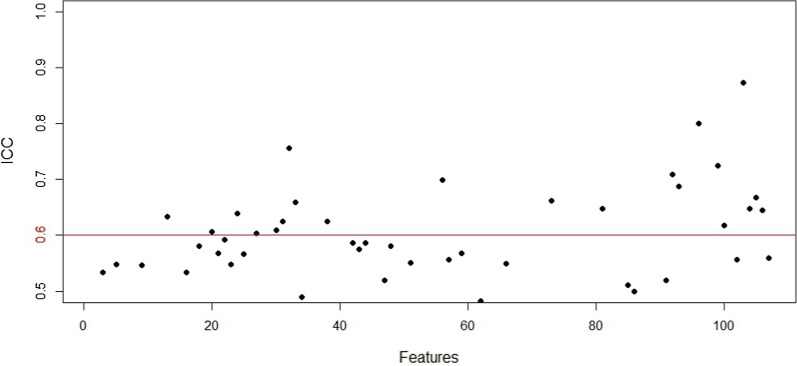
Evaluation of intra-observer’s agreement based on ICC-analysis. 21 of 107 radiomics presented relatively satisfactory agreement(above the red cut-off line)

Results of the intra-observer’s agreement manifested that 19.5% of all 107 features had a relatively satisfactory agreement with ICCs of greater than 0.6 (mean ICC = 0.41, range from 0 to 0.87) (Fig. 4; Table 2). Collected for further analysis.

**Table 2:** native T1-mapping radiomics feature with relatively good intra-agreement

	Features
First-order	RobustMeanAbsoluteDeviation
GLCM	ClusterProminence, Correlation, DifferenceVariance, Idmn, Idn, Imc1, Imc2, MCC
GLDM	SmallDependenceLowGrayLevelEmphasis
GLSZM	GrayLevelNonUniformity, SizeZoneNonUniformity
NGTDM	Contrast, Strength
Shape	Maximum2DDiameterRow, Maximum2DDiameterSlice, MinorAxisLength, Sphericity, SurfaceArea,SurfaceVolumeRatio

### Dimensionality reduction and radiomics feature selection

Univariate analysis results manifested that 21 features were preserved, which were further used for lasso-analysis. 5 features were selected by Lasso-analysis, with best tuned regularization parameter λ of 0.062 under the 1-SE criteria found by tenfold cross-validation. The remaining 5 features were glcm_MCC, glcm_DifferenceVariance, glcm_Imc1, glszm_GrayLevelNonUniformity and shape_MinorAxisLength, respectively (Table 3).

**Table 3:** Performances of differential diagnosis in training and validation set

	AUC (95%CI)	Accuracy (%)	Sensitivity (%)	Specificity (%)
The primary cohort				
LR	0.91(0.84–0.98)	84.1%	73.9%	89.1%
SVM	0.91(0.84–0.98)	84.1%	70.0%	91.3%
Decision tree	0.93(0.86–0.99)	87.0%	73.9%	93.4%
The validation cohort				
LR	0.82(0.67–0.98)	80.0%	70.0%	85.0%
SVM	0.82(0.67–0.98)	80.0%	70.0%	85.0%
Decision tree	0.68(0.49–0.87)	73.3%	50.0%	85.0%

### Radiomics signatures and models

#### The LR model

In the training set, performance of LR reached an AUC of 0.91(95%CI = 0.84–0.98). The sensitivity, specificity and accuracy were 73.9%, 89.1% and 84.1%, respectively. The F1 was 0.76. The AUC was up to 0.82(95%CI = 0.67–0.98) in the validation set. Moreover, the sensitivity, specificity and accuracy were 70.0, 85.0 and 80.0%, respectively. The F1 was 0.70.

#### The SVM model

In the primary cohort, the performance of SVM showed an AUC of 0.91(95%CI = 0.84–0.98), with tuned parameters of gamma = 0.1 and cost = 100. The sensitivity, specificity and accuracy were 70.0, 91.3 and 84.1%, respectively. The F1 was 0.74. In the validation set, the AUC was 0.82(95%CI = 0.67–0.98). The sensitivity, specificity and accuracy were 70.0, 85.0 and 80.0%, respectively. The F1 was 0.70.

#### The tree-decision model

In the primary cohort, the performance of tree-decision showed an AUC of 0.93(95%CI = 0.86–0.99). The sensitivity, specificity and accuracy were 73.9, 93.4 and 87.0%, respectively. The F1 was 0.79. In the validation set, the AUC was 0.68(95%CI = 0.49–0.87). The sensitivity, specificity and accuracy were 50.0, 85.0 and 73.3%, respectively. The F1 was 0.56 (Fig. 4; Table 1.).

## Discussion

In this present study, we developed diagnostic models based on native T1-mapping images to differentiate malignant from benign lesions. According to our results, the SVM and LR classifiers both had satisfactory AUC of 0.82, with sensitivity of 70%, specificity of 85% and accuracy of 80%. The SVM classifier is a powerful tool to analyze data with large number of predictors and limited sample sizes, especially when handling binary outcomes [27]. This is consistence with previous studies [28]. In addition, prior analysis also showed that the LR model based on MRI-radiomics had best performance compared to other classifiers like decision tree, k-nearest neighbor, and XGBoost, etc. [29, 30]. In order to minimize the risk of modeling over-fitting and bias, the LASSO and tenfold cross-validation were used for feature selection and models constructing. In general, our results also manifested that the SVM and LR classifiers, superior to decision tree, are suitable for diagnostic models in indeterminate pulmonary lesions.

At present, the diagnostic work-up of lung cancer using imaging radiomics mainly rely on CT, PET-CT and MRI, of which, CT is the most used. Recently, Gillies, et al. had AUC of 0.80 and 0.85 in discrimination of lung cancer, using size and shape features, non-size based features respectively [31]. Garau et al. found that the handcrafted LDCT radiomics features model (LASSO + SVM) had a higher AUC than the LungRADS clinical model (0.86 vs. 0.76) in the external validation, when identify malignancy [32]. Radiomics features are also valuable in histopathological classification, prognostication, treatment response, and gene mutation, etc. In this study, the radiomics features extracted from native T1-mapping also had clinically significant AUC of 0.82 for malignancy identification. And MRI, being free of radiation dose, is a relatively ideal follow-up inspection tool, especially for women, pregnant women, and people with low immunity. In clinical practice, it is little known about the radiomics features based on MRI modality, mainly T2WI and DWI sequences. Using RFE with SVM, the joint model of T1WI, T2WI, and ADC showed the highest performance with AUC of 0.88 in classification of pulmonary lesions [33]. Besides, radiomics signatures extracted from ADC, DWI, T2WI can be used for predicting EGFR mutation in patients with lung adenocarcinoma [28]. Compared to DWI or IVIM [33], native T1-mapping, obtained in a single breath-holding, had almost no artifacts, deformation and location shift, which is save-timing and more favorable to ensure stability and repeatability of radiomics features. And as far as we know, our team was the first to investigate the value of native T1-mapping radiomics features in differentiating malignant from benign lesions.

Our study showed that the textures features including glcm_MCC, glcm_DifferenceVariance, glcm_Imc1, and glszm_GrayLevelNonUniformity in malignancy were higher than benign lesions, which indicates more significant heterogeneity in malignant lesions. Additionally, the MinorAxisLength in this study had statistically significance in malignancy identification, which could be explained that the lung cancer are usually larger in size than benign lesions. Interestingly, unlike previous report [33], the surface area to volume ratio (SAVR) of lesions was useless in present study. The SAVR presents the degree of a sphere-like shape. We assume that the thickness of the scan sequence has a great influence on the morphology of the lesion. The thinner thickness is more helpful to the value of shape features. Another thing worth mentioning is that there were more benign lesions than malignancy in our samples, which is line with the clinical scenario.

Generally, there are several limitations to this study. Firstly, additional cases were needed for constructing radiomics-based model, which is very important to strengthen the reliability and improve the diagnostic performance. Secondly, the segment of lesions was conducted by one radiologist at a different point in time. The inter-observer’s agreement was absent. Most importantly, this is a single-center experiment and performed on one scanner. Multicenter validation and multi-scanners’ variations still need great effort. Moreover, the effect of image reconstruction and scanners on variability of MRI-based radiomic features needed further investigation. In addition, native T1-mapping failed to identify the mass and secondary obstructive pneumonia. We assumed that post-contrast enhancement T1-mapping would produce better contrast between tumor and background.

## Conclusion

Texture features based on native T1mapping are useful for differentiating pulmonary malignant from benign lesions. The optimal SVM and LR model using 5 texture features acquired the AUC of 0.82, the sensitivity of 70.0%, specificity of 85.0% and accuracy of 80.0%, respectively, in the validation set. Native T1-mapping could be an compensatory tool for the management of pulmonary nodules, especially for those who need long-time follow-ups.

## Data Availability

The datasets generated and/or analyzed during the current study are not publicly available due privacy but are available from the corresponding author on reasonable request.

## Abbreviations

MRI: Magnetic resonance imaging
ICC: Intra-class correlation coefficient
LASSO: Least absolute shrinkage and selection operator
LR: Logical regression
SVM: Support vector machine
AUC: Area under the curve
ROC: Receiver operative characteristic curve
CT: Computed tomography
DWI: Diffusion-weighted imaging
IVIM: Intra-voxel incoherent motion imaging
DCE-MRI: Dynamic-contrast enhanced MRI
StarVIBE: Star-volumetric interpolated breath-hold examination
fBLADE TSE: Fast blade turbo spin echo
FOV: Field of view
ROI: Region of interest
GLCM: Grey-level co-occurrence matrix
GLRLM: Grey-level run-length matrix
GLSZM: Grey-level size-zone matrix
GLDM: Grey-level dependence matrix
NGTDM: Neighbouring gray tone difference matrix
